# MicroRNA profiling of the pubertal mouse mammary gland identifies miR-184 as a candidate breast tumour suppressor gene

**DOI:** 10.1186/s13058-015-0593-0

**Published:** 2015-06-13

**Authors:** Yu Wei Phua, Akira Nguyen, Daniel L. Roden, Benjamin Elsworth, Niantao Deng, Iva Nikolic, Jessica Yang, Andrea Mcfarland, Roslin Russell, Warren Kaplan, Mark J. Cowley, Radhika Nair, Elena Zotenko, Sandra O’Toole, Shi-xiong Tan, David E. James, Susan J. Clark, Hosein Kouros-Mehr, Alexander Swarbrick

**Affiliations:** The Kinghorn Cancer Centre & Cancer Research Division, Garvan Institute of Medical Research, 370 Victoria Street, Darlinghurst, NSW, Sydney, Australia; St Vincent’s Clinical School, Faculty of Medicine, Sydney, UNSW Australia; Cancer Research UK Cambridge Institute, University of Cambridge, Li Ka Shing Centre, Robinson Way, Cambridge, UK; Department of Tissue Pathology and Diagnostic Oncology, Royal Prince Alfred Hospital, Camperdown, NSW Australia; Sydney Medical School, The University of Sydney, Camperdown, NSW Australia; Metabolism in Human Diseases Unit, Institute of Molecular and Cell Biology, A*STAR, 61 Biopolis Drive, Proteos, Singapore; The Charles Perkins Centre, School of Molecular Bioscience, University of Sydney, Camperdown, NSW Australia; Agensys, affiliate of Astellas Pharmaceuticals, 1800 Stewart St, Santa Monica, CA 90403 USA

## Abstract

**Introduction:**

The study of mammalian development has offered many insights into the molecular aetiology of cancer. We previously used analysis of mammary morphogenesis to discover a critical role for GATA-3 in mammary developmental and carcinogenesis. In recent years an important role for microRNAs (miRNAs) in a myriad of cellular processes in development and in oncogenesis has emerged.

**Methods:**

microRNA profiling was conducted on stromal and epithelial cellular subsets microdissected from the pubertal mouse mammary gland. miR-184 was reactivated by transient or stable overexpression in breast cancer cell lines and examined using a series of *in vitro* (proliferation, tumour-sphere and protein synthesis) assays. Orthotopic xenografts of breast cancer cells were used to assess the effect of miR-184 on tumourigenesis as well as distant metastasis. Interactions between miR-184 and its putative targets were assessed by quantitative PCR, microarray, bioinformatics and 3′ untranslated region Luciferase reporter assay. The methylation status of primary patient samples was determined by MBD-Cap sequencing. Lastly, the clinical prognostic significance of miR-184 putative targets was assessed using publicly available datasets.

**Results:**

A large number of microRNA were restricted in their expression to specific tissue subsets. MicroRNA-184 (miR-184) was exclusively expressed in epithelial cells and markedly upregulated during differentiation of the proliferative, invasive cells of the pubertal terminal end bud (TEB) into ductal epithelial cells *in vivo.* miR-184 expression was silenced in mouse tumour models compared to non-transformed epithelium and in a majority of breast cancer cell line models. Ectopic reactivation of miR-184 inhibited the proliferation and self-renewal of triple negative breast cancer (TNBC) cell lines *in vitro* and delayed primary tumour formation and reduced metastatic burden *in vivo*. Gene expression studies uncovered multi-factorial regulation of genes in the AKT/mTORC1 pathway by miR-184. In clinical breast cancer tissues, expression of miR-184 is lost in primary TNBCs while the miR-184 promoter is methylated in a subset of lymph node metastases from TNBC patients.

**Conclusions:**

These studies elucidate a new layer of regulation in the PI3K/AKT/mTOR pathway with relevance to mammary development and tumour progression and identify miR-184 as a putative breast tumour suppressor.

**Electronic supplementary material:**

The online version of this article (doi:10.1186/s13058-015-0593-0) contains supplementary material, which is available to authorized users.

## Introduction

Breast cancer is the most common malignancy that occurs in women globally [1]. Despite the advancement in therapies, many women will suffer from relapse, acquiring metastatic lesions in distant sites and eventually succumbing to cancer related deaths [2–4]. Currently there is a lack of targeted therapies, in particular towards the triple negative breast cancer (TNBC) subtype. TNBC are aggressive and highly invasive, and these tumours lack estrogen receptor (ER), progesterone receptor (PR) and human epidermal growth factor 2 (HER2) expression [5].

A large body of evidence has identified augmented receptor tyrosine kinase (RTK)-PI3K-Akt-mTOR activity in the basal like and TBNCs either due to mutations in RTKs or PIK3CA or loss of phosphatase and tensin homologue (PTEN) expression [6–9]. This pathway has therefore become a major focus of breast cancer drug development, although patient responses to these novel drug compounds in clinical trials have been variable, perhaps due to an incomplete understanding of pathway interactions and feedback loops. Clearly, a thorough understanding of the regulation of these signalling pathways is essential to effectively personalise breast cancer treatment.

microRNAs (miRNAs) are small non coding RNAs that modulate gene expression post transcriptionally. They typically silence their targets by binding their 3′ untranslated regions (UTRs) in a sequence-specific manner [10]. There are a wealth of experimental data demonstrating the pleiotropy of miRNAs in regulating a multitude of cellular processes, such as embryonic stem cell differentiation [11, 12], cell fate and lineage commitment [13–15], organogenesis [16–18] and oncogenesis [19–23].

The pubertal developing mammary gland is elaborated through fat pad invasion by terminal end buds (TEBs); poorly differentiated, unpolarised, proliferative and invasive structures [24] that are enriched for stem and progenitor cell activity [25], with many cellular and molecular similarities to neoplastic cells [26]. We previously examined the expression of mRNAs between cellular subsets of the developing mammary gland, found GATA-3 to be specifically expressed in epithelial cells and went on to identify GATA-3 as an important breast tumour suppressor gene [27, 28].

In this study, we comprehensively profiled miRNA expression in the different cellular compartments within the mammary epithelium of mice. We identify miR-184 as a microRNA associated with epithelial differentiation and demonstrated that miR-184 is silenced and methylated in a subset of TBNC. Further functional characterisation of miR-184 revealed that it is a potential tumour suppressor miRNA in breast cancer; in suppressing cell proliferation, self-renewal in vitro and delaying the formation of metastatic lesions in distant sites in vivo. By performing microarray studies and informatic analysis, we discovered that miR-184 regulates the AKT/mTORC1 pathway by targeting AKT2, TSC2 and PRAS40 in suppressing activity of S6K1 and protein synthesis.

## Methods

### Expression profiling of TEBs, ducts and stroma

TEBs (n = 4), mature ducts (n = 4), and distal stroma regions (n = 4) were microdissected from mammary glands of anaesthetised 5-week-old β-actin–GFP reporter mice (FVB/n, Jackson laboratory, Bar Habor, Maine, USA) using a Leica fluorescence microscope (Leica microsystems, Wetzlar, Germany). Tissue samples were homogenised in Trizol Reagent (Life Technologies, Carlsbad, CA, USA) with a polytron tissue homogeniser (Thermo Fisher Scientific, Waltham, MA, USA). Total RNA was extracted according to a modified protocol based on the manufacturer’s instructions (Invitrogen). The final RNA pellet was ethanol precipitated and washed in 80 % ethanol. RNA samples consisting of TEBs (n = 3), mature ducts (n = 2) and stroma (n = 3) were sent to the Ramaciotti Centre for Gene Function Analysis for miRNA microarray profiling (University of New South Wales, Sydney, Australia) using the SurePrint Mouse miRNA Array V2 (Agilent Technologies, Santa Clara, CA, USA). All animal work was approved by the Garvan/St Vincent’s Hospital Animal Ethics Committee and conducted in accordance with NHMRC guidelines for the ethical treatment of animals.

### Cell lines, retroviral infections

MDA-MB-231 and BT549 cells were maintained in RPMI 1640 supplemented with 10 % FBS and 0.25 % human insulin. HEK293E cells were maintained in DMEM supplemented with 10 % FBS. MDA-MB-231 cells were obtained from the EG & G Mason Research Institute, Worcester, Massachusetts, USA and DNA fingerprinted. BT-549 cell lines were obtained from ATCC. All cell lines used in experiments were cultured at 37 °C in 5 % CO_2_ and 95 % air. MDA-MB-231 cells were first transduced with the retroviral vector pRQ-rtTA-GFP followed by pRQ-miR184.

### Primary tumour samples

Human tumour samples consisting of luminal A (ER+, PR+, Her2−; n = 10), Her2 (ER−, PR−, Her2+; n = 9), triple negative (ER−, PR−, Her2−; n = 7) and matched normal (n = 7) were obtained from the Victoria Cancer Biobank (VCB). Research use of human tissues was approved by the St Vincents Hospital Human Research Ethics Committee (Approval # 08/145). Tumour samples were homogenised using a mortar and pestle. Total RNA samples were extracted using miRVana kit (Invitrogen) and ethanol precipitated with 80 % ethanol.

### Transfection with miRNA mimics

For MDA-MB-231 and BT549 cells, miRIDIAN miRNA mimics (Dharmacon, Lafayette, CO, USA) were transfected into cells using Lipofectamine 2000 (Invitrogen) according to the manufacturer’s protocol for transfecting siRNA. Mimics (final concentration of 50 nM) were mixed with 1 % lipofectamine 2000 (v/v) diluted in Opti-MEM transfection medium (Invitrogen) and incubated for 20 minutes. The mimics were added dropwise onto cells in growth medium at a final concentration of 50 nM. Fresh medium was replaced 24 h post transfection. A non-radioactive cell proliferation assay (Promega, Madison, WI, USA) was used to assess the number of viable cells. Three biological replicates were conducted.

MDA-MB-436 and HS578T breast cancer cells were reverse transfected with 40 nM of either miR-184 mimic or scrambled using Dharmafect 4 reagent (Dharmacon) following manufacturer’s instructions; SiTox (Dharmacon) was used as a positive control. The following day, medium was changed and cells were left in culture for an additional 72 h. To assess viability, CellTiter-Glo (Promega) was added directly into the cell medium (1:2 ratio) and left to incubate for 10 minutes; luminescence was read using FLUOstar Omega plate reader (BMG Labtech, Ortenburg, Germany). The experiment was performed in three biological replicates.

### Quantitative RT-PCR

Total RNA was extracted using the Trizol reagent (Invitrogen) method with a modification where the final RNA pellet was ethanol precipitated and washed in 80 % ethanol. cDNA was generated using the Taqman MiRNA Reverse Transcription Kit (Applied Biosystems) using the specific primer from TaqMan MiRNA assay (Applied Biosystems) according to the manufacturer’s protocol. Quantitative PCR (qPCR) amplification was run on the 7900 Real-time PCR system (Applied Biosystems). All human and mouse miRNA expression values were normalised to RNU6B and SnoRNA202, respectively.

### Immunoblot and 3′ UTR luciferase activity assay

Cells were lysed using complete radioimmune precipitation assay (RIPA) buffer supplemented with complete ULTRA protease inhibitor cocktail tablets (Roche, Basel, Switzerland) and sodium orthovanadate. Anti-Akt2, anti-phospho-Akt (Thr308), anti-phospho-Akt (Ser473), anti-Pras40, anti-phospho-Pras40 (Thr246), anti-Gsk3A, anti-phospho-Gsk3 (Ser21/9), anti-Tsc2, anti-phospho-Tsc2 (Thr1426), anti-mTOR, anti-phospho-mTOR (Ser2448), anti-p70S6k1, anti-phospho-p70S6k1 (Thr389), anti-p70S6K2, anti-4E-BP1, anti-4E-BP1 (Thr37/46) (Cell Signalling Technology, Danver, Massachusetts, USA) rabbit polyclonal antibodies were used in immunoblotting. Luciferase constructs (pLightSwitch_3′UTR) (Switchgear genomics, Carlsbad, CA, USA) containing the 3′ UTR region of Akt2, Pras40, Gsk3a, CSF1 and Itgb1 was individually transfected into HEK293T cells using pGL4.12 (luc2CP) as a normaliser. Luciferase activity was measured by using the dual luciferase assay (Promega).

### Tumoursphere assays

MDA-MB-231 cells were cultured in serum-free RPMI 1640, supplemented with B27 (Invitrogen) and 20 ng/ml bFGF (BD Biosciences, Franklin Lakes, NJ, USA), and 4 μg/ml heparin (Sigma Aldrich, St. Louis, MO, USA) and plated at 15,000 viable cells/well in ultralow attachment 6-well plates (Corning Incorporate, NY, USA). Complete serum-free medium was added to the cells every 3 days. Primary tumourspheres were enumerated at day 10. Primary tumourspheres were collected, and were enzymatically dissociated into single cells, re-plated in ultralow attachment 6-well plates (Corning Incorporate) at a density of 1,000 viable cells/well and enumerated at day 10.

### Protein synthesis assay

Cells were washed twice and serum starved for 16–18 h prior to EGF stimulation. Cells were stimulated with EGF in serum-free DMEM low glucose without L-ARG, L-LEU, L-LYS, sodium pyruvate and phenol red (Sigma Aldrich) for 1 h: [^3^H]Leucine (PerkinElmer, Waltham, MA, USA) was added at the same time as EGF to a final concentration of 5 μCi/ml. Cells were washed three times in ice-cold PBS, lysed using RIPA buffer followed by incubating cells with 10 % trichloroacetic acid (TCA) for 10 minutes to precipitate proteins. Pellets were washed three times in 10 % TCA. Pellets were resuspended in 50 nM NaOH with 1 % Triton X-100 at 65 °C for 30 minutes or until the pellet dissolved. The radioactivity of samples was assessed by measuring the scintillation count using the β-scintillation counter. The results were normalised for protein content using bicinchoninic acid (BCA) analysis.

### Animal experiments

For primary tumour burden and spontaneous metastasis assays, 1 × 10^6^ MDA-MB-231 cells were injected into the mammary fat pad of 8-week-old female NOD/SCID mice. Mice were culled at the ethical end point, and primary tumour and other organs such as lungs, spleen, lymph node, pancreas and brain were harvested. Metastatic lesions were quantified with a fluorescent microscope within 2 h of harvest.

### Immunohistochemistry

Mouse tissues were extracted and fixed overnight at 4 °C in 10 % neutral-buffered formalin (Sigma), and stored in 70 % ethanol at 4 °C. Subsequently, tissues were embedded in paraffin and sectioned. Sections were stained with haematoxylin and eosin (H&E) and phospho-histone H3 (Cell signaling) in accordance with standard protocols. Scoring of phospho-Histone H3 immunostaining and mitotic figures was assessed by a specialist breast pathologist (SO’T).

### Gene expression analysis

MDA-MB-231 cells were transfected with miR-184 or control mimics for 48 h before total RNA was extracted using the modified Trizol reagent protocol with an additional ethanol precipitation step. RNA samples were sent off to the Ramaciotti Centre for Gene Function Analysis for gene expression profiling using the affymetrix gene 1.0ST array (Affymetrix) (University of New South Wales, Sydney, Australia). Gene expression analysis was performed using gene pattern.

### Statistical analysis

Statistical analysis was performed by using GraphPad Prism v6.0. T tests were performed to determine statistical significance, unless otherwise stated. *P* <0.05 was considered statistically significant.

### Methylation analysis

The MBDCap-Seq experiment was performed by Dr Claire Stirzaker and Dr Jenny Song (Garvan Institute of Medical Research). Analysis of the results was performed by Dr Elena Zotenko (Garvan Institute of Medical Research). Briefly, methylated DNA was isolated using the MethylMinerTM Methylated DNA Enrichment Kit (Life Technologies). Genomic FFPET DNA was sonicated. MBD-Biotin Protein (3.5 μg) was coupled to 10 μl of Dynabeads M-280 Streptavidin according to the manufacturer’s instructions.

MBD biotin conjugated to the magnetic beads was washed three times and resuspended in one volume of 1 × bind/wash buffer. The capture reaction was performed by adding 500 ng to 1 μg sonicated DNA to the MBD-magnetic conjugates on a rotating mixer for 1 h at room temperature (RT). All capture reactions were done in duplicate. The beads were washed three times with 1 × bind/wash buffer. The bound methylated DNA was eluted using single high-salt elution buffer (2 M NaCl). Eluted DNA fraction was concentrated by ethanol precipitation using 1 μl glycogen (20 μg/μl), 1/10 volume of 3 M sodium acetate, pH 5.2 and two sample volumes of 100 % ethanol, and resuspended in 60 μl water.

### Preparation of MBDCap-Seq libraries and Illumina sequencing

DNA, 10 ng, was prepared for Ilumina sequencing using the Illumina ChIP-Seq DNA sample prep kit (Illumina, San Diego, CA, USA) according to the manufacturer’s instructions. The library preparation was analysed on Agilent High Sensitivity DNA 1000 Chip. Each sample was sequenced on one lane of the GA11x.

### Alignment of MBDCap-Seq data

Sequenced reads were aligned to the hg18 version of the human genome with bowtie [29]. Sequence reads with three mismatches or more and reads mapping to multiple positions were excluded. Last, multiple reads mapping to exactly the same genomic coordinate were eliminated and only one read was retained for downstream analysis.

### miRNA seed match analysis

The seed match analysis was performed as previously described by Melton et al. [30]. Briefly, ensemble transcripts (hg19) of promoter, 5′ UTR, open reading frame (ORF) and 3′ UTRs and other annotated genes (hg19) were obtained from the UCSC Genome Browser. Relevant miRNA seed match (7mer-1A or 7mer-m8) was conducted on those transcripts using a custom Python script [30]. Results from seed match analysis were mapped to Affymetrix IDs. Wilcoxon rank sum test was used to determine the *p* values in this analysis.

### Gene signature score and survival analysis

A stringent 18-gene signature repressed by miR-184 (fold-change >2, Table 2) was assessed for survival analysis using two independent cohorts from METABRIC [31] and a cohort of women receiving neo-adjuvant chemotherapy [32]. METABRIC gene expression data were downloaded from the European Genome-Phenome Archive (EGAS00000000083). Gene expression and clinical data from Hatzis et al. were downloaded from Gene Expression Omnibus (GEO) [GEO: GSE25066]. The gene signature score was defined by a weighted average method [33] for each sample in the METABRIC discovery cohort. Survival curves were estimated using the Kaplan-Meier method, with overall survival used as the outcome metric.

## Results

### Identification of microRNAs enriched in mammary cellular compartments

**Fig. 1:**
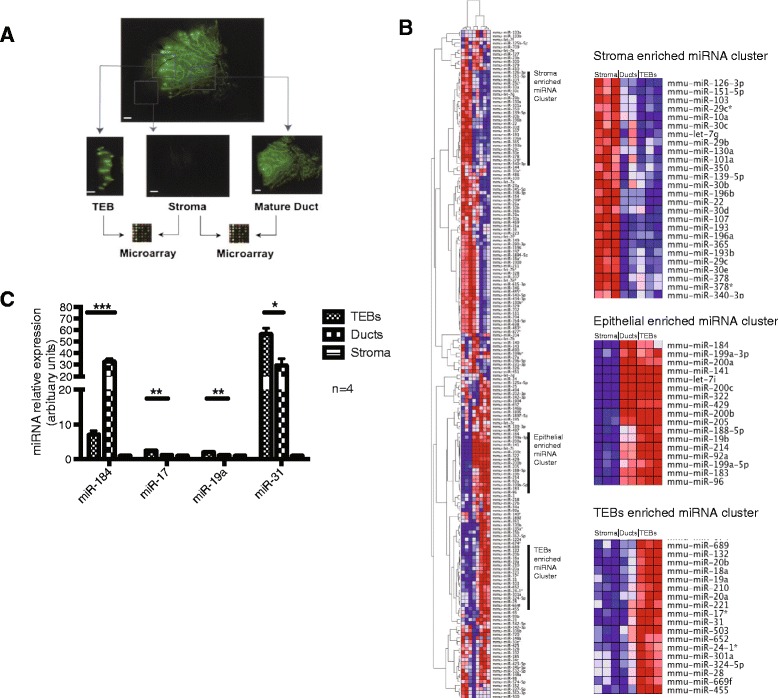
Differential microRNA expression by mammary cellular subsets. **a** Micro-dissection of terminal end buds (*TEBs*) and mature ducts from 5-week-old GFP+ mice before performing miRNA expression profiling. **b** Unsupervised hierarchical clustering of microRNAs reveals groups of microRNAs with tissue-specific expression. **c** Validation of candidate microRNA expression using stemloop quantitative RT-PCR. The *t* test was performed: *p <0.05 indicates significant difference comparing candidate miRNA expression in TEBs with mature ducts

We performed miRNA expression profiling on micro-dissected stroma, mature ducts and TEBs of pubertal 5-week-old GFP+ mice to identify miRNAs involved in mammary gland development (Fig. 1a). Each cellular fraction expressed a set of unique microRNAs (Fig. 1b). We identified a set of miRNAs that were specific to the stroma, ducts, TEBs or both epithelial fractions (Fig. 1b; Table 1). miR-31 was the most highly enriched miRNA expressed in the TEBs, approximately 10.7-fold upregulated versus ducts. When we performed unsupervised hierarchical clustering (Fig. 1b), and miR-31 was tightly clustered to several members of the proto-oncogenic miR 17–92 cluster, such as miR-17, miR-18a and miR-19a. Conversely, miR-184 expression was significantly enriched approximately 4.2-fold in the mature ducts compared to the TEBs. Interestingly, miR-184, being the most highly enriched miRNA in the mature ducts was clustered tightly to a subset of epithelial specific miRNAs, which included members of the miR-183 family (miR-183, miR-96) and all members of the miR-200 family (miR-141, miR-200a, miR-200b, miR-200c and miR-429). We validated the expression of miR-31, miR-184, miR-17 and miR-19a in TEBs and ducts by quantitative RT-PCR (Fig. 1c).

**Table 1:** Candidate microRNAs enriched in the terminal end buds (TEBs) or mature ducts, ranked by fold change

Rank	Upregulated	microRNA candidate	microRNA family	*Q* value	Fold change (TEB/duct)
1	TEB	mmu-miR-31	miR-31	0.009	10.76
2	TEB	mmu-miR-17*	miR-17	0.019	7.81
3	TEB	mmu-miR-18a	miR-17	0.005	4.72
4	TEB	mmu-miR-362-5p	miR-362	0.027	4.62
5	TEB	mmu-miR-19a	miR-17	0.007	3.27
1	Duct	mmu-miR-184	miR-184	0.01	0.24
2	Duct	mmu-miR-7 g*	Let-7	0.028	0.3
3	Duct	mmu-miR-1894-5p	miR-1894	0.027	0.37
4	Duct	mmu-miR-346	miR-346	0.021	0.39
5	Duct	mmu-miR-328	miR-328	0.024	0.41

### miR-184 expression is attenuated in mouse models of breast cancer and human breast cancer cell lines

To examine whether microRNAs differentially regulated through morphogenesis are also deregulated in cancer, we examined miRNA expression in four mouse tumour models compared to normal total mammary epithelium: spontaneous *Tp53*^*−/−*^ tumours [34], transgenic C3 SV40 tag model [35], transgenic MMTV-Neu [36] and transgenic MMTV-PyMT [37]. There are several advantages of using these tumour models: first, each tumour model has been well characterised and the initiating oncogene that drives tumourigenesis in these models is known. Second, each tumour model has its matched normal counterpart as a comparison. Last, these tumour models recapitulate different subtypes of human breast cancer.

**Fig. 2:**
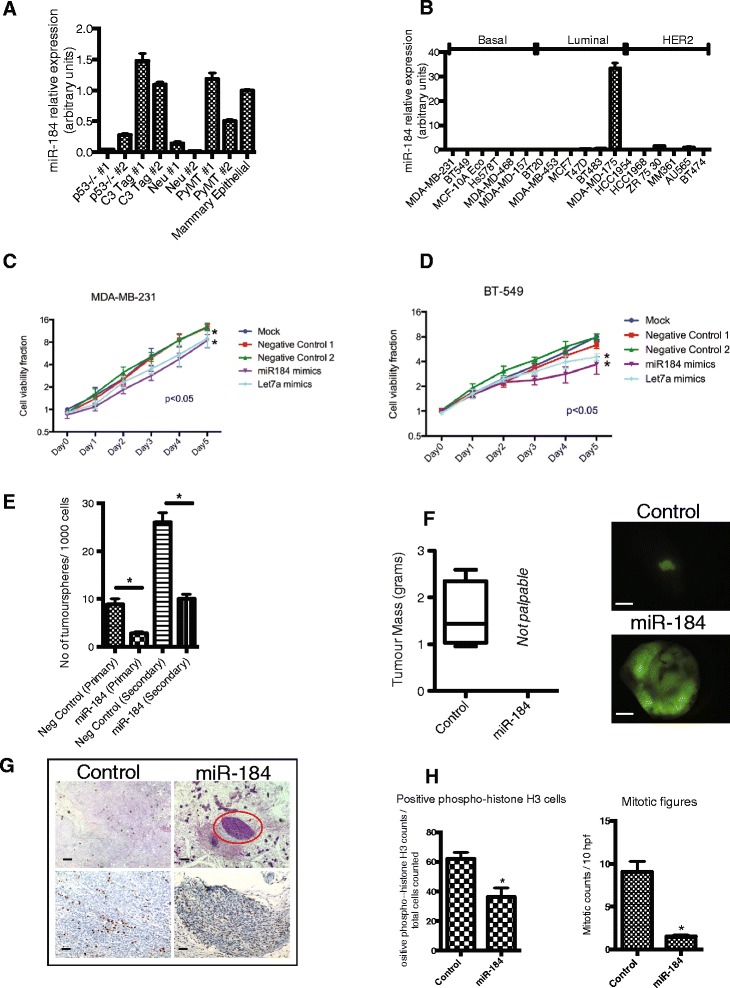
Expression of miR-184 is lost in cancer models. Ectopic expression suppresses proliferation and self-renewal in vitro and in vivo. **a** miR-184 expression is lost in several mouse models of breast cancer, compared to total mouse mammary epithelium. microRNA expression was normalised to SnoRNA202. **b** miR-184 is undetectable in a majority of human breast cancer cell lines. microRNA expression is normalised to RNU6. **c** MDA-MB-231 were transfected with microRNA mimics, proliferation was measured for 5 days by MTS assay. **d** BT-549 were transfected with microRNA mimics, proliferation was measured for 5 days using MTS assay. **c**, **d** Results are expressed as mean + standard error from three independent experiments performed in sextuple technical replicates. The *t* test was performed: *p <0.05 indicates significant difference in proliferation in cells overexpressing miR-184 compared with non-targeting miRNA control. **e** MDA-MB-231 stably overexpressing miR-184 and control were cultured in low adherent plates and enumerated for primary and secondary tumourspheres. The *t* test was performed: *p <0.05 indicates significant difference in tumoursphere-forming potential in cells overexpressing miR-184 compared to control. **f** Tumour mass in NOD/SCID mice (n = 5) after injection of MDA-MB-231 constitutively overexpressing miR-184, let-7a or negative control into the mammary fat pad. **g** Haematoxylin and eosin (H&E) (*top*) and phospho-histone H3 (*bottom*) immunohistochemical analysis. *Circle* identifies focus of MDA-MB-231 cells at the injection site. *Top scale bars* = 50 μM, *bottom scale bars* = 30 μM. **h** Quantitation of phospho-histone H3 immunoreactivity and mitotic figures in tumour sections. The *t* test was performed: *p <0.05 indicates significant difference in positive phospho-histone H3 expression and mitotic figures in miR-184 cohort compared to control cohort. *HER2* human epithelial growth factor receptor 2, *neg* negative

miR-31 was highly expressed in all of the murine tumours when compared to the normal murine mammary epithelial cells (Additional file 1: Figure S1D). In contrast, the expression of miR-184 was almost abrogated in the *Tp53*^*−/−*^ tumours and the MMTV-Neu tumours, however, miR-184 expression in the C3 SV40 Tag and MMTV-PyMT tumours was comparable to normal mammary epithelial cells suggesting that miR-184 might be specifically silenced in certain breast cancers or that they derive from different cells of origin (Fig. 2a). In addition, we also examined miR-17 and miR-19a, in these murine tumours. Despite the known role of this microRNA cluster in oncogenesis, there was no enrichment of these two miRNAs in murine tumours when compared to the normal mammary epithelial cells (Additional file 1: Figure S1E, F).

We interrogated the relative expression of miR-31, miR-184, miR-17 and miR-19a in a panel of human breast cancer cell lines. Seven basal breast cancer cell lines comprising both basal A and basal B and five luminal cell lines were chosen to represent these breast cancer subtypes [38]. The expression of miR-31 was restricted to the basal breast cancer cell lines compared to the luminal cell lines (Additional file 1: Figure S1A). miR-17 and miR-19a were relatively evenly expressed across cell lines (Additional file 1: Figure S1B, C). Conversely, the expression of miR-184 was undetectable in a majority of cell lines with the exception of MDA-MB-175 (Fig. 2b).

### miR-184 suppresses proliferation of human breast cancer cell lines in two-dimensional and suspension culture

To elucidate its function in cancer, miR-184 was acutely overexpressed in highly proliferative human breast cancer cell lines: MDA-MB-231, BT-549, MDA-MB-436 and HS578T, which express undetectable levels of endogenous levels of miR-184. Let-7a is a well-characterised tumour suppressor miRNA and has been shown to impede proliferation in cancer cell lines, and hence, was used as a positive control in this experiment. The exogenous overexpression of miR-184 resulted in levels comparable to the endogenous levels of miR-184 detected in the MDA-MB-175 (not shown). miR-184 inhibited cell proliferation significantly in all four models (Fig. 2c, d, Additional file 1: Figure S1G). The positive control let-7a also exhibited an anti-proliferative effect, which was of a similar magnitude to the result obtained with miR-184 (Fig. 2c, d).

Given the increase in expression of miR-184 as mammary epithelia differentiated in vivo, we asked whether ectopic miR-184 expression regulates the self-renewal capacity of breast cancer cells. Tumoursphere assays [39] were performed in MDA-MB-231 cells constitutively overexpressing miR-184. This assay requires cells to be in cultured in suspension for an extended period, therefore, miR-184 was overexpressed via retroviral transduction. MDA-MB-231 cells overexpressing miR-184 had a 50 % reduction in tumoursphere-forming potential in primary tumourspheres as well as in secondary tumourspheres when compared to the negative control (Fig. 2e).

### miR-184 suppresses tumour initiation and proliferation in vivo

After ascertaining the inhibitory effect of miR-184 on proliferation and self-renewal in vitro, we wanted to examine the role of miR-184 in vivo. Control and miR-184 overexpressing MDA-MB-231 cells were separately injected into the mammary fat pad of cohorts of immunocompromised NOD/SCID mice (n = 5). At 10 weeks post transplantation, primary tumours and internal organs were harvested. All the mice in the control group developed solid tumours, however, in stark contrast, none of the mice within the miR-184 cohort had developed any palpable tumours at this time point (Fig. 2f). Under detailed visual examination of the miR-184 cohort by fluorescent microscopy, a small population of GFP+ cells could be detected in the mammary gland. H&E staining identified a bolus of miR-184 overexpressing cells localised to the injection site (Fig. 2g). These results suggested that cells overexpressing miR-184 have impaired proliferation in vivo.

We performed immunohistochemical staining on the tumour sections for phospho-histone H3 and scoring for mitotic figures, both of which measure actively dividing cells. A significantly lower proportion of cells overexpressing miR-184 were positive for phospho-histone H3 positive cells and mitotic figures compared to the control group (Fig. 2h), confirming the suppression of proliferation by miR-184.

### miR-184 expression prolongs survival and reduces metastatic burden

**Fig. 3:**
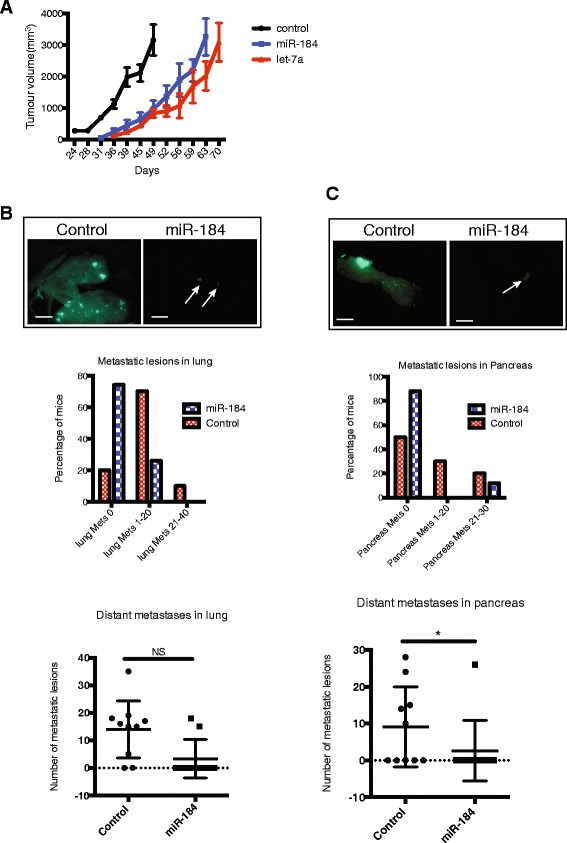
miR-184 delays onset of metastases (*mets*) in distant organs and prolongs survival. **a** Growth rate of tumours after injection of MDA-MB-231 into the mammary fat pad in NOD/SCID mice (n = 10 per group) constitutively overexpressing miR-184, let-7a or negative control. **b** Representative images of micrometastases in the lung and percentage of NOD/SCID mice (n = 10) with 0, 1–20 or 21–40 metastatic lesions in the lung. **c** Pancreas of control and miR-184 cohorts and percentage of NOD/SCID mice (n = 10) with 0, 1–20 or 21–30 metastatic lesions in the pancreas. The *t* test was performed; *p <0.05 indicates significant difference in the number of micrometastases in miR-184 cohort compared to control cohort. Scale bar = 200 μM. *NS* not significant

We conducted a survival experiment to ascertain if miR-184 expression extended survival. Mice were transplanted with MDA-MB-231 cells expressing miR-184, Let-7a or negative control and aged to ethical endpoint. The control group developed palpable tumours as early as 24 days post transplantation, and these tumours propagated at a faster rate when compared to the miR-184 group (Fig. 3a). In contrast, there was delayed latency in tumour growth in the miR-184 group where mean time to tumour palpation was by day 38. This delay in tumour initiation was also observed in the let-7a cohort and translated to an increase in overall survival. This result suggests that miR-184 impedes tumour initiation and growth.

Lastly, we also examined if miR-184 could reduce metastatic burden in vivo. Visual examination and quantification by fluorescence microscopy at ethical endpoint was used to identify macroscopic metastases in the lung and pancreas, the most frequent sites of metastasis in this model. The majority of control mice developed multiple metastases in the lungs and pancreas. In contrast, only approximately 20 % of mice within the miR-184 cohort developed any metastatic lesions in the lung (*p* = 0.06) (Fig. 3b). In addition, we also observed a similar reduction in metastatic burden in the pancreas, where only 10 % of mice from the miR-184 cohort developed any pancreatic metastases (Fig. 3c).

### Regulatory targets of miR-184

Next, we aimed to identify the repertoire of miR-184 targets to explain the impact of miR-184 on proliferation. We capitalised on the evidence that miRNAs can destabilise the mRNA of their targets to use a gene-expression-based approach to target identification [40]. We transfected miR-184 or control microRNA mimics into MDA-MB-231 breast cancer cells and analysed global gene expression changes compared to controls by affymetrix gene arrays.

**Fig. 4:**
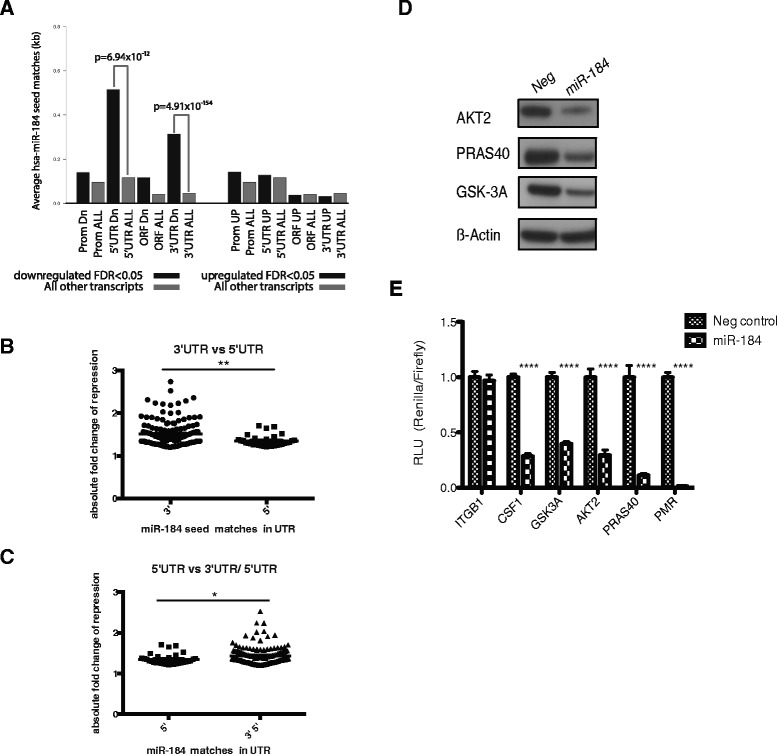
miR-184 represses various substrates in the AKT/mTOR signaling pathway. **a** Analysis of miR-184 seed matches in the promoter, 5′ UTR, open reading frame (ORF) and 3′ UTR of miR-184 downregulated and upregulated transcript reveals strong enrichment for miR-184 seed matches in the 5′ and 3′ UTRs of downregulated genes. The Wilcoxon rank-sum test was used to calculate *p* values. **b** Distribution of gene expression changes in genes with miR-184 seed regions in either 3′ UTR or 5′ UTR only. **c** Distribution of gene expression changes in genes with miR-184 seed regions in either 5′ UTR or 3′ and 5′ UTR only. **d** Immunoblot of total AKT2, PRAS40, GSK3A and Actin in MDA-MB-231 transfected with negative (*neg*) control or miR-184 mimics for 48 h. **e** Luciferase 3′ UTR reporter assay performed in HEK293T confirmed that CSF1, GSK3A, AKT2 and PRAS40 are direct targets of miR-184. ITGB1 is a negative control and a reporter containing 3′ UTR sequences perfectly complementary to mIR-184 (PMR) serves as a positive control

We observed 1,263 mRNAs and 592 mRNAs that were significantly upregulated and downregulated respectively in cells overexpressing miR-184 compared with the negative control, suggesting dramatic remodelling of gene expression by miR-184 expression. From the profiling data, we filtered and identified the top miR-184 repressed mRNA targets based on their fold change (Table 2). *AKT1S1*, more commonly termed *PRAS40* was the most significantly downregulated gene with approximately 3.1-fold change, followed by *LAT1*, which was repressed approximately 2.7-fold. To identify direct targets of miR-184 from the profiling data, we adopted a comprehensive seed match analysis method previously described by Melton et al. [30]*,* which interrogates the promoter, 5′ UTR, CDS and 3′ UTRs of transcripts downregulated following microRNA overexpression for the presence of miR-184 seed matches. The analysis revealed highly significant enrichment of miR-184 seed matches within the 5′ UTR and 3′ UTR regions of the downregulated transcripts, suggesting that miR-184 modulates its targets by targeting the UTRs (Fig. 4a). We did not observe any significant enrichment of miR-184 seed regions within the upregulated genes, suggesting that this method identifies bone fide direct targets.

**Table 2:** Top 30 genes significantly repressed by miR-184 (Q <0.05)

Genes	Fold change	*Q* value
*PRAS40*	3.1092	0.0000887
*SLC7A5*	2.7426	0.0001686
*CSF1*	2.5244	0.00004997
*RRP1B*	2.3603	0.00006787
*SEMA7A*	2.3251	0.0013856
*ADAM19*	2.3148	0.00003501
*TNFSF18*	2.3009	0.0005973
*CARM1*	2.2891	0.00004997
*POM121*	2.2415	0.00006787
*SH3GL1*	2.2386	0.00003501
*S100A16*	2.2311	0.00003501
*KLC2*	2.2168	0.00003501
*PPAP2B*	2.1852	0.00007764
*PLBD2*	2.1415	0.00003501
*CYB5R3*	2.0392	0.00006787
*LASP1*	2.0308	0.00003501
*PPP6R1*	2.0175	0.00003501
*CDC25A*	2.0013	0.0004932
*PNO1*	1.9322	0.0011347
*HAS2*	1.9214	0.0014053
*SEPT9*	1.9143	0.0001192
*MYADM*	1.9084	0.0001448
*PLAGL2*	1.9068	0.00019
*GSK3A*	1.902	0.00006787
*AKT2*	1.9014	0.00004997
*REXO1*	1.8962	0.0004027
*FSCN1*	1.8733	0.0004771
*STK40*	1.8697	0.00008406
*IL7R*	1.8555	0.0064985
*TMEM45A*	1.8489	0.0064985

Of the 557 downregulated genes, 193 of the genes possessed a miR-184 seed match in the UTR; of these 193 genes, 135 of them had a miR-184 seed match only in the 3′ UTR, 30 genes contained a miR-184 seed match only in the 5′ UTR and 23 genes possessed a miR-184 seed region in both the 3′ and 5′ UTR. The remaining genes contained miR-184 binding sites in either the CDS and/or UTRs. We asked whether the number of miR-184 seed matches within the downregulated genes correlated with a greater degree of repression. Interestingly, in contrast to previous studies [41, 42], we observed no additive or synergistic effect on mRNA destabilisation when multiple seed matches were detected (Additional file 2: Figure S2A). We also saw no added enrichment in repression when there was multiple miR-184 seed match regions located only in the 3′ UTR (Additional file 2: Figure S2B) or the 5′ UTR (Additional file 2: Figure S2C) or in both the 3′ and 5′ UTR (Additional file 2: Figure S2D). Genes with miR-184 seed match in their 3′ UTR were also significantly more downregulated compared to those with a miR-184 seed match in the 5′ UTR (Fig. 4c). These data indicate that miR-184 represses many of its mRNA targets by targeting the 3′ UTR and that the number of miR-184 binding sites within the 3′ UTR does not correlate with degree of mRNA destabilisation.

Our analysis of putative direct targets identified at least 158 targets that are repressed and contain a 3′ UTR seed match. To look for functional relationships between these putative targets, we applied gene set enrichment analysis (GSEA) [43]. There was an enrichment of genes involved in oxidative stress, PI3K/AKT signalling, apoptosis via NFKB and axon repulsion (Additional file 2: Figure S2E). We focused on the PI3K/AKT pathway for two reasons. First, AKT2 has been shown to be an miR-184 direct target in neuroblastoma [44]. Second, AKT signalling is often dysregulated in breast cancer and *AKT2* has been identified as a driver gene in mammary tumourigenesis [45].

Within the PI3K/AKT gene list, there was core enrichment for several genes from our profiling data (marked as *yes* under the core enrichment column), suggesting that the differential expression of these genes was significant (Additional file 3: Table S1). AKT2, PRAS40 and GSK3A were immunoblotted to validate the microarray result. There was a marked reduction in the total protein expression of AKT2, PRAS40 and GSK3A when miR-184 was overexpressed (Fig. 4d). In order to ascertain whether these genes were targets of miR-184, HEK293T cells were transfected with miR-184 or negative control in combination with 3′ UTR luciferase reporters for *CSF1, GSK3A, AKT2* or *PRAS40* in addition to *ITGB1* (used as a negative control) and a construct containing perfect matches to the miR-184 sequence as a positive control (PMR). There was no repression in the luciferase activity of *ITGB1* and a near complete ablation in luciferase activity of the PMR. We observed robust repression (>50 %) in the luciferase activity of reporters carrying the 3′ UTR from *CSF1, GSK3A, AKT2* and *PRAS40* signifying that miR-184 expression can act via the 3′ UTR to destabilise the mRNA of these targets (Fig. 4e).

### miR-184 targets AKT/mTOR protein synthesis pathway

AKT is a central node for orchestrating myriad pro-survival and proliferative pathways in oncogenesis [46]. One such downstream effector pathway is the mTOR signalling cascade. The mTOR pathway consists of two mTOR complexes, mTORC1 and mTORC2. The mTORC1 complex comprises RAPTOR, mLST8 and PRAS40 and when activated, initiates cell growth, proliferation and protein synthesis by regulating S6K1 and 4E-BP1 [47]. From the GSEA, there was enrichment for targets genes such as TSC2, RPS6KB2, implicated in mTORC1 mediated protein synthesis in cells overexpressing miR-184, and hence, we asked if miR-184 could be an important regulator of AKT/mTORC1 protein synthesis pathway.

**Fig. 5:**
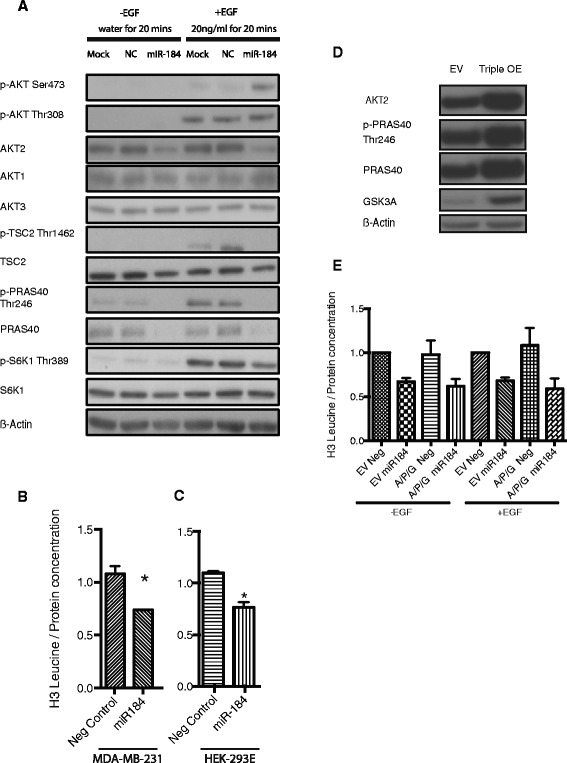
miR-184 suppresses protein synthesis by negatively regulating AKT/mTORC1 pathway. **a** Immunoblots of members of the AKT/mTOR pathway in MDA-MB-231 transfected with miR-184 mimics and treated with and without epidermal growth factor (*EGF*). **b** Measurement of protein synthesis by using B-Scintillation in MDA-MB-231 transfected with negative control or miR-184 mimics for 24 h, serum starved overnight and treated with labelled H3 leucine. **c** Measurement of protein synthesis by using B-Scintillation in HEK293E transfected with negative control or miR-184 mimics for 24 h, serum starved overnight and treated with labelled H3 leucine. **d** Western blot confirming overexpression of AKT2, PRAS40 and GSK3A. **e** Protein synthesis in cells overexpressing AKT2 (**a**), PRAS40 (P) and GSK3A or control (empty vector, *EV*), transfected with miR-184 mimics (184) or controls (*Neg*)

We transfected MDA-MB-231 cells with miR-184 mimics, serum starved them and stimulated the cells with EGF, which is known to initiate a cascade of signalling events to promote cell proliferation, growth and survival. As expected, there was a marked decrease in total AKT2 and PRAS40 expression in cells overexpressing miR-184 and no change in the total protein expression of AKT1 and AKT3. Interestingly, miR-184 also decreased the total protein expression of mTOR (Additional file 4: Figure S3A). In control cells treated with EGF, we observed activation of AKT as both Ser473 and Thr308 were phosphorylated (Fig. 5a). In return, activated AKT inhibited the activities of TSC2 and PRAS40 through phosphorylating them at Thr1462 and Thr246 respectively (Fig. 5a), thus initiating the activation of mTORC1. Conversely when cells overexpressing miR-184 were treated with EGF, we observed a modest increase in p-AKT Thr308, indicating that there was increased AKT activity. Despite the increased AKT activity, the inhibition on TSC2 was completely abrogated, as observed from the dephosphorylation of Thr1462, possibly by miR-184 targeting AKT2. Finally the activating phosphorylation of S6K1 at Threonine-389, a rate-limiting factor in protein synthesis, was attenuated without affecting the activity of 4E-BP1 (Additional file 4: Figure S3A).

We also further examined if this miR-184-regulated signalling cascade was recapitulated in vivo. Within the cohort of mice bearing miR-184-overexpressing tumour cells, the expressions of a number of miR-184 targets (e.g., AKT2, GSK3A and S6K2) were reduced in three out of four mice when compared to the control tumours. Furthermore, we also observed a dramatic repression of mTOR expression accompanied by the dephosphorylation of S6K1 indicating a partial attenuation of the protein synthesis pathway (Additional file 4: Figure S3B).

To directly assay the impact of EGF stimulation and miR-184 expression on protein synthesis, we transfected MDA-MB-231 cells with the miR-184 mimics, and measured the amount of protein synthesised in the cell by incorporating radiolabelled tritiated (H^3^) leucine into cells in the presence of EGF. miR-184 repressed global protein synthesis dramatically (Fig. 5b). In order to demonstrate that this regulatory phenomenon exhibited by miR-184 was not restricted to the MDA-MB-231 model, we performed the same assay with the HEK293E cells using another growth factor, insulin. We detected that miR-184 similarly repressed global protein synthesis by approximately 30 % in the HEK293E cells stimulated with insulin (Fig. 5c).

### Overexpression of AKT2, PRAS40 or GSK3A does not rescue miR-184 repression of protein synthesis

We were interested to see if we could reverse the repression on protein synthesis by overexpressing some of the core enriched top candidate genes identified from the GSEA. Therefore we tested if direct targets AKT2, PRAS40 or GSK3A were responsible for the effect of miR-184 on MDA-MB-231 cell protein synthesis by stably overexpressing AKT2, PRAS40 and GSK3A individually or in combination, together with transfection of miR-184 or negative control mimics. Despite the overexpression of these direct target genes individually (Additional file 5: Figure S4A, B, C) or in combination (Fig. 5d, e), protein synthesis was nonetheless suppressed by miR-184 expression in these cell lines. These results suggest that miR-184 regulates the protein synthesis process by modulating expression of genes in addition to AKT2, PRAS40 and GSK3A.

### miR-184 expression and prognostic significance in breast cancer

**Fig. 6:**
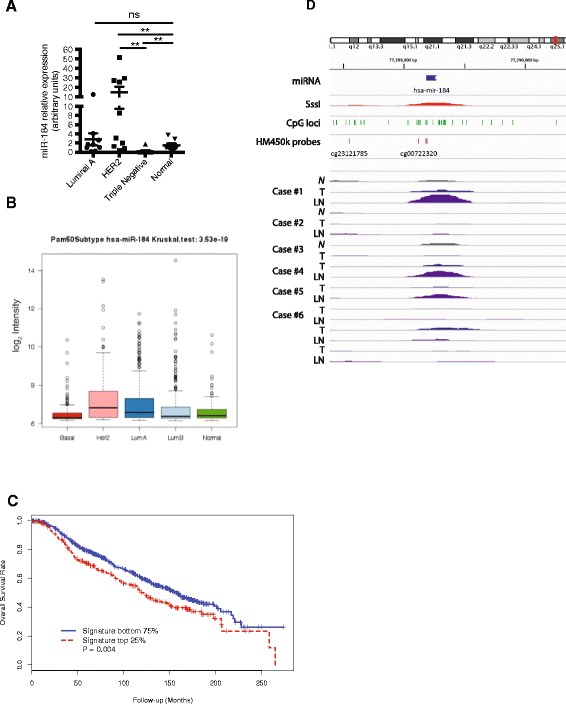
miR-184 is downregulated in triple negative breast cancer, methylated in metastatic lesions and reduced expression of stringent miR-184 targets correlates with poor overall survival in breast cancer patients. **a** miR-184 expression in luminal A, human epidermal growth factor (*HER2*), triple negative and matched normal tissue. The *t* test was performed: *p <0.05, **p <0.01. **b** Analysis of miR-184 expression in the Molecular Taxonomy of Breast Cancer International Consortium (METABRIC) patient cohort stratified by intrinsic subtype: *p* <1 × 10^−18^. **c** Kaplan-Meier survival analysis comparing the outcome of METABRIC patients stratified by signature score of miR-184 repressed targets (*red*, samples with top 25 % signature score (n = 246); *blue*, samples with bottom 75 % signature score (n = 734)). Overall survival was used as the outcome metric. **d** The miR-184 locus is hypermethylated in some metastatic lymph node biopsies (*LN*) when compared to primary tumour (*T*) and normal breast tissue (*N*). SssI MBD2IP (*red*) has been treated with methylase and acts as a positive control for methylation at this locus

We next investigated the significance of miR-184 expression in breast cancer and its association with clinico-pathological measures. We measured miR-184 expression in snap-frozen primary tumour specimens comprising patient samples diagnosed with luminal, HER2-amplified and triple negative cancers and matched normal tissue. We observed unique expression patterns for miR-184 across these subtypes. There were no significant differences in the expression of miR-184 between luminal cancers and matched normal. However within the HER2 subtype, the patients fell into two groups: normal-like and high miR-184 expression. Last, miR-184 expression was significantly lower in the triple negative subtype compared to the matched normal (Fig. 6a). To verify this result in a bigger patient cohort, we analysed miR-184 expression in the METABRIC cohort of 980 breast cancers [31] and also observed that miR-184 mean expression was highest in HER2-positive breast cancers and significantly lower in basal breast cancers, a subset of TNBC (Fig. 6b). In this cohort, miR-184 expression did not correlate with prognosis (data not shown). However, elevated expression of a signature composed of high-confidence direct targets of miR-184 predicted poor overall survival (Fig. 6c). This effect was observed in two further independent cohorts (Additional file 6: Figure S5). In multivariate analysis, this signature predicted poor prognosis independent of ER status in two out of three cohorts (Additional file 7: Table S2). These data are consistent with a role for miR-184 in suppressing proliferation or metastatic dissemination.

### miR-184 is epigentically silenced in human cancers

There is emerging evidence for epigenetic silencing by hypermethylation [48] of miRNAs with tumour suppressor properties or growth inhibitory functions in various malignancies [49–52]. Methyl-capture sequencing was performed to capture a global snapshot of the genome-wide DNA methylation profile of normal mammary tissue, primary TNBC tumours and matched metastatic tumours micro-dissected from the lymph nodes of patients. We observed that there was minimal methylation detected at the miR-184 locus in normal breast tissue. In contrast, there was a pronounced increase in methylation in metastatic tumours in the lymph nodes of three of eight patients (Fig. 6d), suggesting a selective pressure to silence miR-184 during metastatic dissemination, consistent with the capacity of miR-184 to suppress metastasis in animal models (Fig. 3).

## Discussion

In our study, we have discovered miRNAs enriched in different subcellular compartments of the developing mammary ductal structure. These results suggest that some miRNAs are expressed in specific subcellular compartments, such as the TEBs and mature ducts, to modulate cellular processes such as proliferation and differentiation during ductal elongation. We then asked, however, if these miRNAs were differentially expressed and functional in cancer. As a proof of concept, we found TEB-enriched miRNAs to be highly expressed in a panel of breast cancer models whereas miRNAs enriched in the ducts displayed an opposite trend, being lowly expressed in cancer.

One of the miRNAs that followed this expression pattern was miR-184. Upon functional characterisation, there was compelling evidence to suggest that miR-184 is a tumour suppressor in certain cancer subtypes, as it suppressed cell proliferation and self-renewal in vitro and tumour growth in the primary and distant sites. A role in modulating metastasis is supported by analysis of a subset of TNBC patient samples, where miR-184 was epigenetically silenced in lymph node metastases, suggesting silencing of miR-184 can promote metastatic dissemination. Upon interrogating a large breast cancer cohort (METABRIC) dataset, we also observed a significant decrease in miR-184 expression in the ER-negative tumours compared to the ER-positive tumours. In this cohort, very few microRNAs associate with prognosis [53], as we found for miR-184. However, elevated expression of high-confidence targets repressed by miR-184 predicted poor prognosis, consistent with our evidence from animal models that elevated miR-184 activity improves outcome.

miR-184 is crucial in regulating certain developmental processes such as the differentiation of neural stem cells, germ line cells and corneal epithelial cells [54–56]. Several studies have established that miR-184 is lowly expressed in different malignancies such as childhood neuroblastoma, brain cancers, clear cell renal cell carcinoma and prostate cancer [44, 57–60]. When miR-184 was ectopically overexpressed in vitro, it resulted in cell cycle arrest and apoptosis [44, 57, 58], as well as impeding neuroblastoma xenograft formation resulting in longer survival in vivo [61]. Though several downstream targets of miR-184 such as AKT2, NUMBL, SHIP2, NFAT1 have been identified in different cell types, nevertheless there have been no definite reports on the functional role of miR-184 in breast cancer nor detailed analysis of signalling pathways that are potentially modulated by miR-184 [44, 54, 62, 63].

miR-184 has been previously shown to be controlled by epigenetic mechanisms in development. This was identified in the mouse brain, where the genomic region proximal to the miR-184 locus in adult neural stem cells contains CpG rich sequences instead of canonical CpG islands. Methyl-CpG binding protein 1 (Mbd1) binds to these CpG-rich sequences in the genomic regions surrounding miR-184, and represses the transcriptional activity of miR-184 [54]. In a separate study, researchers performed bisulphite sequencing on umbilical cord blood graft CD4+ T cells and discovered a small putative CpG island just upstream of the miR-184 locus, and an additional 32 CpG sites present within the adjacent regions of the miR-184 locus making it an ideal target for epigenetic silencing [63].

We are the first to provide evidence to suggest that the attenuated expression of miR-184 in cancer is potentially a result of epigenetic mechanisms. miR-184 was methylated in a subset of lymph node metastases in TNBC, providing supportive evidence that miR-184 may play a role as a novel mammary tumour suppressor. We hypothesised that the methylation of miR184 in metastatic tissue suggests a selective pressure against maintenance of miR-184 particularly during metastatic dissemination.

Our experimental evidence suggests that the anti-tumourigenic properties displayed by miR-184 are a consequence of miR-184 inhibiting the activity of the PI3K/AKT/mTORC1 pathway, therefore limiting protein synthesis. This study suggests that miR-184 suppresses the protein synthesis pathway by targeting several important members of the AKT/mTOR pathway. miR-184 represses the total levels of AKT2, which relieves the inhibitory function on TSC2, the crucial negative regulator of the mTORC1 pathway. In addition, the reactivation of TSC2 results in the abrogation of S6K1 activity, the effector of the protein synthesis pathway.

Furthermore, this signalling event was partially recapitulated in vivo, where miR-184 repressed several substrates within the AKT/mTORC1 pathway in a majority of tumours. The total expression of mTOR was reduced in the miR-184 cohort, potentially inhibited the formation of the mTORC1 and therefore reducing the activity of S6K1. Interestingly, we also observed a loss of TSC2 expression and an increase in PRAS40 expression in the miR-184 cohort. It is possible that these changes are compensation to circumvent the anti-proliferative effects of miR-184 during tumour progression.

These changes in the signalling pathway correlate with our protein synthesis assay, where we observe less synthesised proteins in cells overexpressing miR-184. However, we do not fully comprehend how miR-184 suppresses protein synthesis. Despite the overexpression of AKT2, PRAS40 and GSK3A in combination, we were unable to rescue the miR-184 mediated suppression of protein synthesis and hence more work needs to be conducted to define the rate limiting targets in protein synthesis and proliferation downstream of miR-184.

Several other targets may explain this phenotype. *S6K2*, a member of 40S ribosomal protein S6 kinase family was also repressed by miR-184 at the mRNA and protein level. The 40S ribosomal S6 kinase is a direct substrate of mTORC1 signalling, and when activated it drives cell growth and proliferation by recruiting translational machineries and initiating protein production in cells [64]. Even though S6K2 is homologous to S6K1, sharing 83 % identical amino acid sequences, they both display unique functions [65, 66].

*CSF1* and *LAT1* were also significantly repressed when miR-184 was overexpressed. Evidence in the literature associates both genes with promoting tumourigenesis and metastasis [67–77]. LAT1 also plays an important role in transporting available amino acids into the cell for protein synthesis, which provides a possible explanation for the inability to revert the defect in protein synthesis even by the overexpression of AKT2, PRAS40 and GSK3A.

As the PI3K/AKT/mTOR axis is such a crucial signalling axis in mediating a myriad of cellular functions essential in both normal development and during carcinogenesis, many research groups have focussed on elucidating the convoluted regulation of these pathways. In recent years, studies have revealed that there is a landscape of microRNAs that specifically regulate various components of the PI3K/AKT/mTOR signalling axis in orchestrating a series of fundamental cellular processes in normal development; including stem cell expansion [78], wound healing [79] and smooth muscle and pancreatic beta cell proliferation [80, 81].

## Conclusions

We propose that miR-184 mimics the role of a capacitor to control signalling current through the PI3K-mTOR pathway in the presence of a stimulus by targeting intermediates of the AKT/mTOR cascade and in turn tuning the signalling output. Integration of miR-184 expression and activity in PI3K-AKT-mTOR pathway analysis may help better predict pathway dynamics and response to therapeutics targeting this pathway.

## Additional files

Additional file 1: Figure S1. Validation of differential microRNA expression and additional human cell line transfections. miR-31 (**A**), miR-17 (**B**) and miR-19a (**C**) expression in a panel of breast cancer cell lines. microRNA expression was normalised to RNU6. miR-31 (**D**), miR-17 (**E**), and miR-19a (**F**) expression in murine tumour models. microRNA expression was normalised to SnoRNA202 (G0 miR-184 suppresses proliferation in MDA-MB-436 and HS578T cells in vitro. Graphs depict the mean +/− standard error of the mean of three independent experiments; **p* <0.05. Additional file 2: Figure S2. MicroRNA target identification. **A** Total number of miR-184 seed matches in the promoter, 5′ UTR, open reading frame (ORF) and 3′ UTR of downregulated transcripts. **B** Total number of miR-184 seed matches present only in the 3′ UTR of downregulated transcripts. **C** Total number of miR-184 seed matches present only in the 5′UTR of downregulated transcripts. **D** Total number of miR-184 seed matches present only in both 3′ and 5′ UTR of downregulated transcripts. **E** Gene set enrichment analysis of miR-184. Enrichment plots and statistics are shown for the nine most significantly (false discovery rate <0.05) downregulated gene sets involved in oxidative stress, trabectedin resistance, PI3K/AKT, apoptosis via NFKB, and axon repulsion. Additional file 3: Table S1. miR-184 modulates the activity of a number of gene targets within the PI3K/AKT pathway. Core enrichment of gene targets potentially regulated by miR-184 is represented by *Yes* under the core enrichment column. Additional file 4: Figure S3. miR-184 suppresses protein synthesis by negatively regulating certain substrates in AKT/mTORC1 pathway. **A** Immunoblots of members of AKT/mTOR pathway in MDA-MB-231 transfected with miR-184 mimics and treated with and without epidermal growth factor (*EGF*). **B** Immunoblots of members of AKT/mTOR pathway in primary tumour lysates derived from xenografts of mDA-MB-231 cells overexpressing miR-184 or control, harvested at ethical end point. Additional file 5: Figure S4. Overexpression of miR-184 direct targets does not rescue the suppression of protein synthesis. Measurement of protein synthesis by using B-scintillation in MDA-MB-231 cells overexpressing AKT2 (**A**), PRAS40 (**B**), GSK3A (**C**) or in combination transfected with negative control or miR-184 mimics for 24 h, serum starved and treated with labelled H3 leucine in the absence and presence of epidermal growth factor (*EGF*). Additional file 6: Figure S5. Reduced expression of stringent miR-184 targets correlates with poor overall survival in two independent cohorts of breast cancer patients. Kaplan-Meier survival analysis comparing outcome of patients stratified by signature score of miR-184 repressed targets. *Red*: samples with top 25 % signature score; *blue*: samples with bottom 75 % signature score. Overall survival was used as the outcome metric. **A** Molecular Taxonomy of Breast Cancer International Consortium (METABRIC) validation cohort (n = 248 (*red*), n = 745 (*blue*). **B** Cohort from Hatzis et al. [31], n = 128 (*red*), n = 380 (*blue*). Additional file 7: Table S2. Univariate and multivariate analysis of prognostic associations for miR184 signature.

## Abbreviations

ATCC: American type culture collection
BCA: bicinchoninic acid
bFGF: bovine fibroblast growth factor
cDNA: complementary deoxyribonucleic acid
CDS: coding sequence
CSF1: colony stimulating factor 1
DMEM: Dulbecco’s modified Eagle’s medium
EGF: epidermal growth factor
ER: estrogen receptor
FBS: fetal bovine serum
GATA-3: GATA binding protein 3
GEO: Gene expression omnibus
GSEA: gene set enrichment analysis
GSK3A: glycogen synthase kinase 3A
H&E: haematoxylin and eosin
HER2: human epidermal growth factor 2
ITGB1: integrin B1
MBD: methyl-CpG-binding domain
METABRIC: Molecular Taxonomy of Breast Cancer International Consortium
miRNA: MicroRNA
mLST8: mammalian lethal with Sec13 protein 8
MMTV: mouse mammary tumour virus
mRNA: messenger RNA
mTORC1: mechanistic target of rapamycin complex
NOD: non-obese diabetic
ORF: open reading frame
PBS: phosphate-buffered saline
PCR: polymerase chain reaction
PI3K: phosphatidylinositol 3-kinase
PIK3CA: phosphatidylinositol-4,5-bisphosphate 3-kinase, catalytic subunit alpha
PR: progesterone receptor
PRAS40: proline rich AKT substrate 40 kDa
PTEN: phosphatase and tensin homologue
PyMT: polyoma middle T-antigen
qPCR: quantitative polymerase chain reaction
RIPA: radioimmune precipitation assay
RNU6B: RNA, U6 small nuclear
RPS6KB1: ribosomal protein S6 kinase, 70 kDa, polypeptide 1
RTK: receptor tyrosine kinases
rtTA: reverse tetracycline transactivator
SCID: severe combined immunodeficiency
SLC7A5: solute carrier family 7 (amino acid transporter light chain, L system), member 5
TCA: trichloroacetic acid
TEBs: terminal end buds
TNBC: triple negative breast cancer
TSC1/2: tuberous sclerosis 1/2
UTR: untranslated region
VCB: Victoria Cancer Biobank
