# Supplementary figures and images for: MicroRNA profiling of the pubertal mouse mammary gland identifies miR-184 as a candidate breast tumour suppressor gene
Source: Breast Cancer Res. 2015 Jun 13;17(1):83. doi: 10.1186/s13058-015-0593-0 (PMC4504458; doi:10.1186/s13058-015-0593-0)

**A**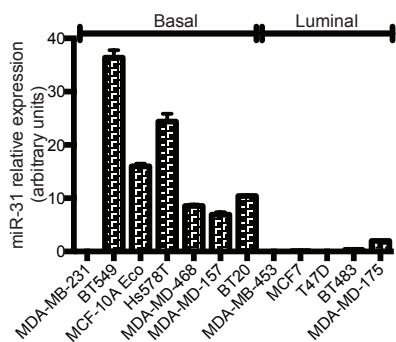**B**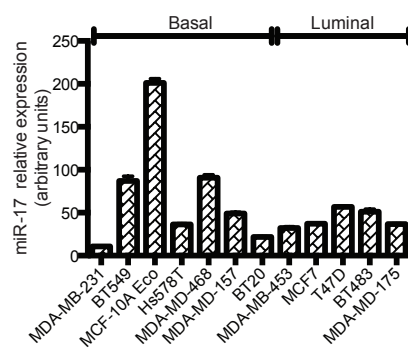**C**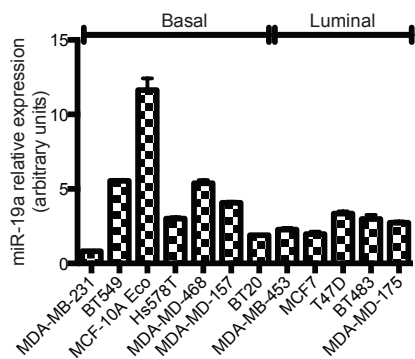**D**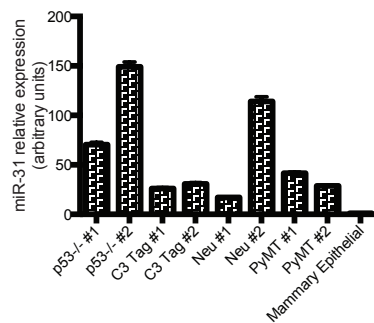**E**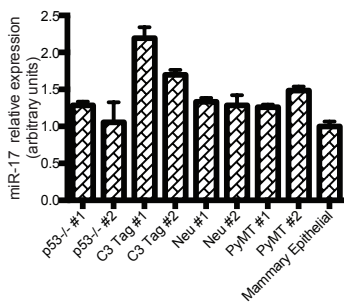**F**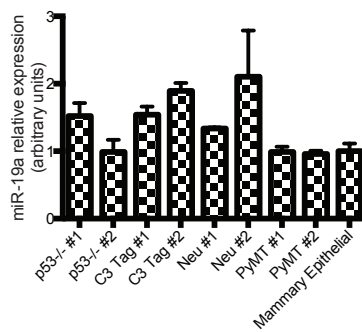**G**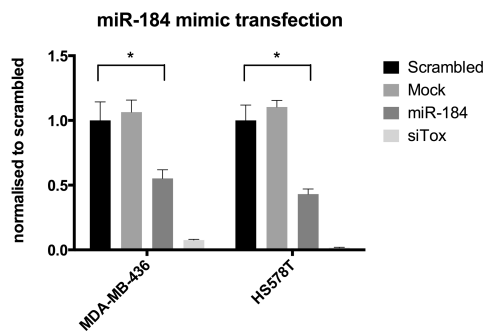

Supplement: Additional file 1: Figure S1. — Validation of differential microRNA expression and additional human cell line transfections. miR-31 (A), miR-17 (B) and miR-19a (C) expression in a panel of breast cancer cell lines. microRNA expression was normalised to RNU6. miR-31 (D), miR-17 (E), and miR-19a (F) expression in murine tumour models. microRNA expression was normalised to SnoRNA202 (G0 miR-184 suppresses proliferation in MDA-MB-436 and HS578T cells in vitro. Graphs depict the mean +/− standard error of the mean of three independent experiments; *p <0.05. [file 13058_2015_593_MOESM1_ESM.pdf]

A

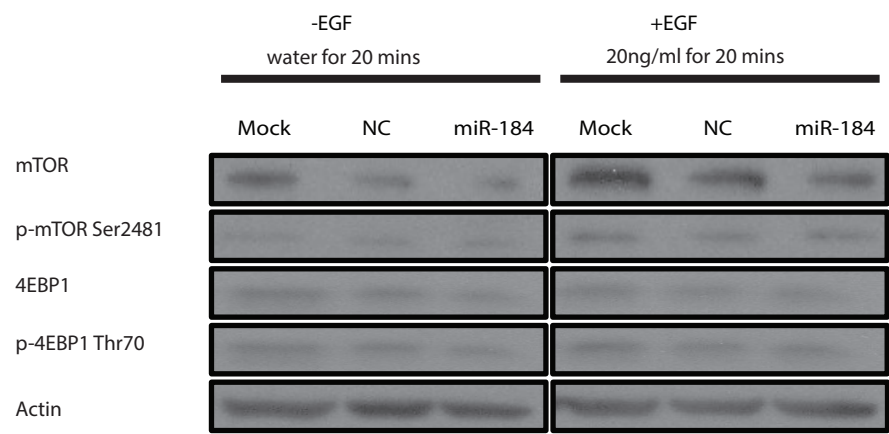

B

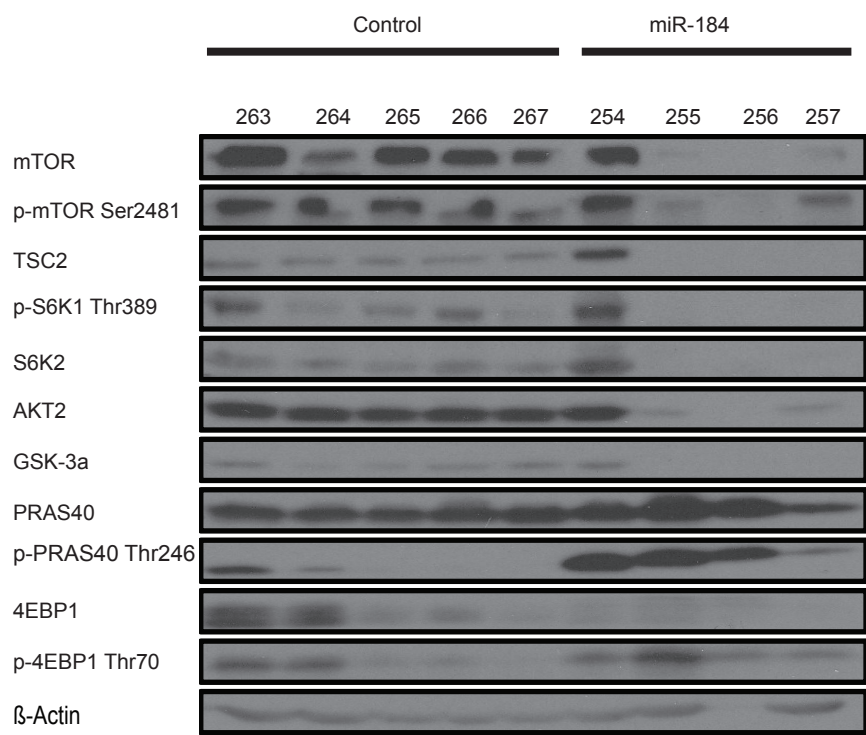

Supplement: Additional file 4: Figure S3. — miR-184 suppresses protein synthesis by negatively regulating certain substrates in AKT/mTORC1 pathway. A Immunoblots of members of AKT/mTOR pathway in MDA-MB-231 transfected with miR-184 mimics and treated with and without epidermal growth factor (EGF). B Immunoblots of members of AKT/mTOR pathway in primary tumour lysates derived from xenografts of mDA-MB-231 cells overexpressing miR-184 or control, harvested at ethical end point. [file 13058_2015_593_MOESM4_ESM.pdf]

A

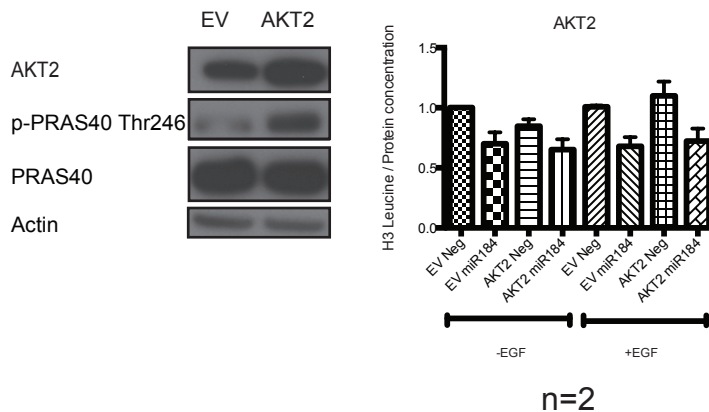

B

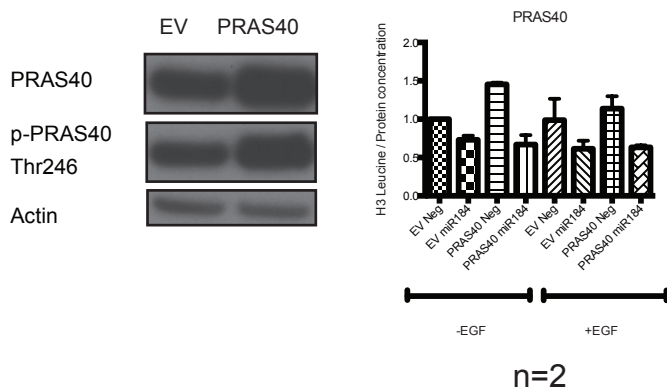

C

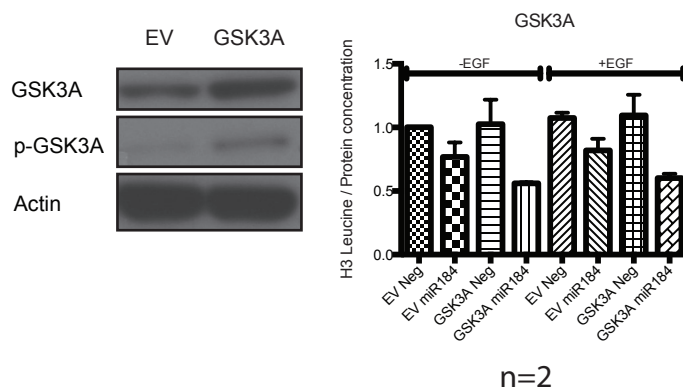

Supplement: Additional file 5: Figure S4. — Overexpression of miR-184 direct targets does not rescue the suppression of protein synthesis. Measurement of protein synthesis by using B-scintillation in MDA-MB-231 cells overexpressing AKT2 (A), PRAS40 (B), GSK3A (C) or in combination transfected with negative control or miR-184 mimics for 24 h, serum starved and treated with labelled H3 leucine in the absence and presence of epidermal growth factor (EGF). [file 13058_2015_593_MOESM5_ESM.pdf]

A

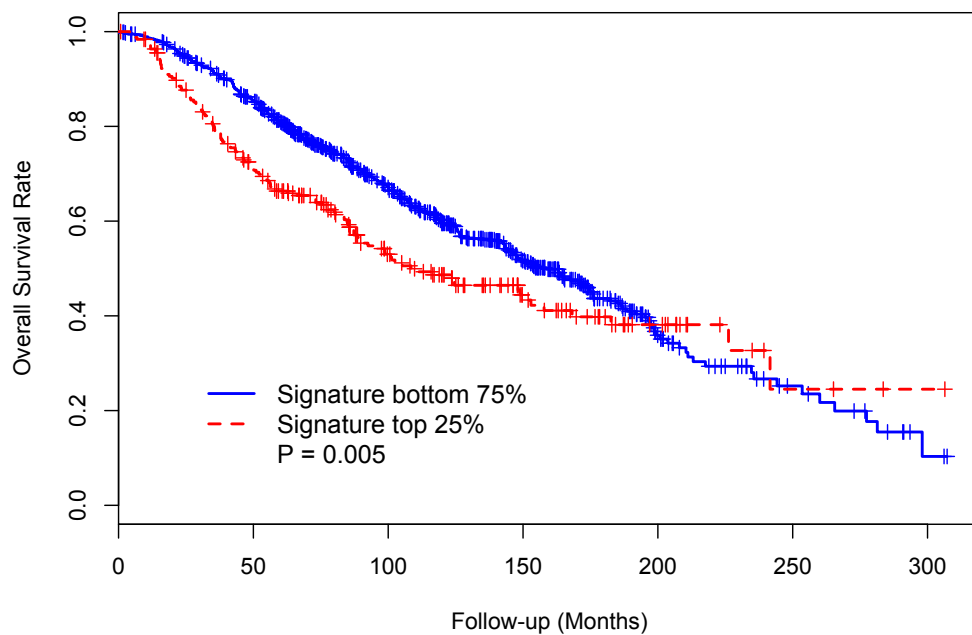

B

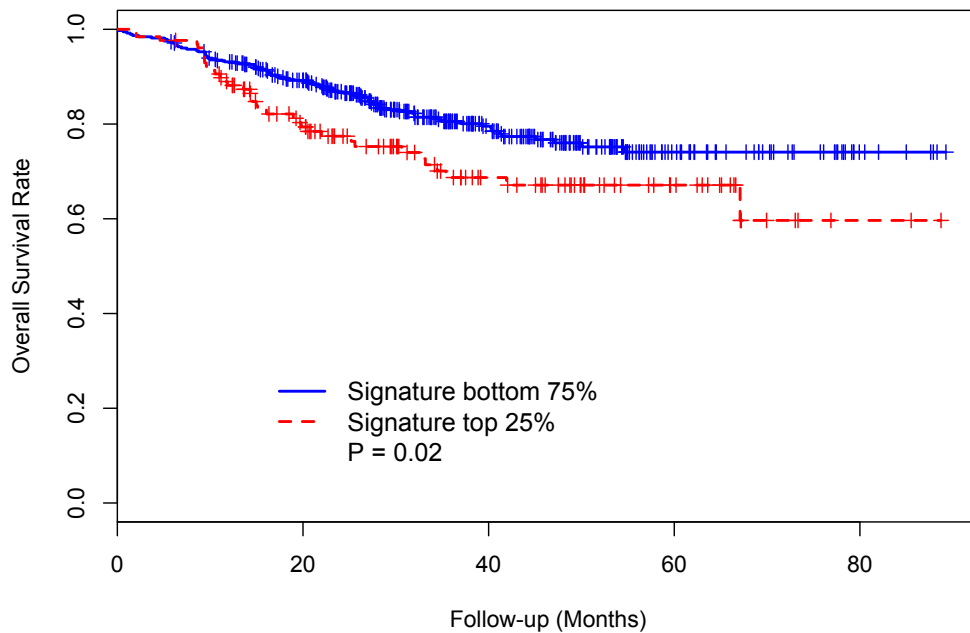

Supplement: Additional file 6: Figure S5. — Reduced expression of stringent miR-184 targets correlates with poor overall survival in two independent cohorts of breast cancer patients. Kaplan-Meier survival analysis comparing outcome of patients stratified by signature score of miR-184 repressed targets. Red: samples with top 25 % signature score; blue: samples with bottom 75 % signature score. Overall survival was used as the outcome metric. A Molecular Taxonomy of Breast Cancer International Consortium (METABRIC) validation cohort (n = 248 (red), n = 745 (blue). B Cohort from Hatzis et al. [31], n = 128 (red), n = 380 (blue). [file 13058_2015_593_MOESM6_ESM.pdf]
