# Supplementary material for: MicroRNA profiling of the pubertal mouse mammary gland identifies miR-184 as a candidate breast tumour suppressor gene
Source: Breast Cancer Res. 2015 Jun 13;17(1):83. doi: 10.1186/s13058-015-0593-0 (PMC4504458; doi:10.1186/s13058-015-0593-0)
Supplement: Additional file 2: Figure S2. — MicroRNA target identification. A Total number of miR-184 seed matches in the promoter, 5′ UTR, open reading frame (ORF) and 3′ UTR of downregulated transcripts. B Total number of miR-184 seed matches present only in the 3′ UTR of downregulated transcripts. C Total number of miR-184 seed matches present only in the 5′UTR of downregulated transcripts. D Total number of miR-184 seed matches present only in both 3′ and 5′ UTR of downregulated transcripts. E Gene set enrichment analysis of miR-184. Enrichment plots and statistics are shown for the nine most significantly (false discovery rate <0.05) downregulated gene sets involved in oxidative stress, trabectedin resistance, PI3K/AKT, apoptosis via NFKB, and axon repulsion. [file 13058_2015_593_MOESM2_ESM.pdf]

A

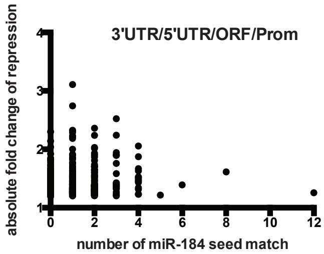

B

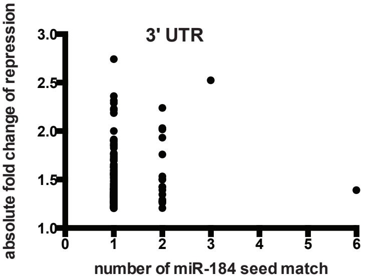

C

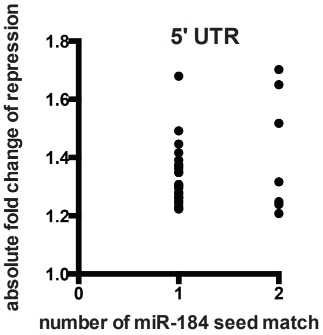

D

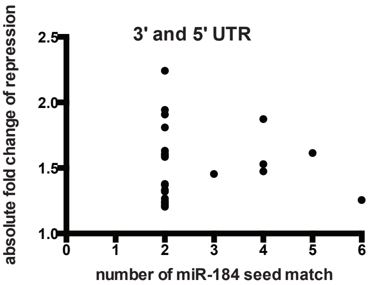

E

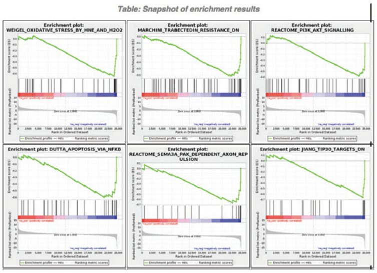

Enrichment plot	NES	FDR Q-VALUE
WEIGEL_OXIDATIVE_STRESS_BY_HNE_AND_H2O2	-2.20	0.009
MARCHINI_TRABECTEDIN_RESISTANCE_DN	-2.12	0.022
REACTOME_PI3K_AKT_SIGNALLING	-2.04	0.041
DUTTA_APOPTOSIS_VIA_NFKB	-2.04	0.033
REACTOME_SEMA3A_PAK_DEPENDENT_AXON_REPULSION	-2.03	0.028
JIANG_TIP30_TARGETS_DN	-2.03	0.024
SNIJDERS_AMPLIFIED_IN_HEAD_AND_NECK_TUMOURS	-2.02	0.023
WONG_ENDMETRIUM_CANCER_UP	-1.98	0.037
BENPORATH ES CORE NINE CORRELATED	-1.96	0.041
