# Supplementary material for: MicroRNA profiling of the pubertal mouse mammary gland identifies miR-184 as a candidate breast tumour suppressor gene
Source: Breast Cancer Res. 2015 Jun 13;17(1):83. doi: 10.1186/s13058-015-0593-0 (PMC4504458; doi:10.1186/s13058-015-0593-0)
Supplement: Additional file 3: Table S1. — miR-184 modulates the activity of a number of gene targets within the PI3K/AKT pathway. Core enrichment of gene targets potentially regulated by miR-184 is represented by Yes under the core enrichment column. [file 13058_2015_593_MOESM3_ESM.pdf]

Gene symbol	Core enrichment
PIK3CB	No
GSK3B	No
RICTOR	No
CASP9	No
NTRK1	No
PIK3R1	No
CDKN1B	No
PDPK1	No
AKT3	No
CDKN1A	No
MTOR	No
CREB1	No
PTEN	No
CHUK	No
BAD	No
PHLPP1	No
FOXO4	No
FOXO3	No
NGF	No
NR4A1	No
AKT1	No
RHOA	No
MLST8	No
THEM4	No
FOXO1	No
TRIB3	No
IRS2	Yes
MAPKAP1	Yes
IRS1	Yes
PIK3CA	Yes
PIK3R2	Yes
TSC2	Yes
MDM2	Yes
RPS6KB2	Yes
AKT1S1	Yes
GSK3A	Yes
AKT2	Yes
