# Supplementary material for: MicroRNA profiling of the pubertal mouse mammary gland identifies miR-184 as a candidate breast tumour suppressor gene
Source: Breast Cancer Res. 2015 Jun 13;17(1):83. doi: 10.1186/s13058-015-0593-0 (PMC4504458; doi:10.1186/s13058-015-0593-0)
Supplement: Additional file 7: Table S2. — Univariate and multivariate analysis of prognostic associations for miR184 signature. [file 13058_2015_593_MOESM7_ESM.pdf]

Hatzisetal Cohort

Table S2A Unitivariate analysis of prognostic association for mir184 signature

Model 1 (Predictors: mir184 signature)	Hazard Ratio (95% CI)	P-value
mir184 signature high vs mir184 signature low	1.581 (1.062, 2.353)	**0.024**

Table S2B Multivariate analysis of prognostic associations for mir184 signature and ER status

Model 2 (Predictors: mir184 signature and ER status)	Hazard Ratio (95% CI)	P-value
mir184 signature high vs mir184 signature low	1.175 (0.777, 1.777)	0.445
ER IHC positive vs ER IHC negative	0.357 (0.240, 0.532)	**3.86E-07**

METABRIC Discovery Cohort

Table S2C Unitivariate analysis of prognostic association for mir184 signature

Model 3 (Predictors: mir184 signature)	Hazard Ratio (95% CI)	P-value
mir184 signature high vs mir184 signature low	1.350 (1.098, 1.659)	**0.0044**

Table S2D Multivariate analysis of prognostic associations for mir184 signature and ER status

Model 4 (Predictors: mir184 signature and ER status)	Hazard Ratio (95% CI)	P-value
mir184 signature high vs mir184 signature low	1.294 (1.050, 1.595)	**0.016**
ER IHC positive vs ER IHC negative	0.731 (0.583, 0.917)	**0.0068**

METABRIC Validation Cohort

Table S2E Unitivariate analysis of prognostic association for mir184 signature

Model 5 (Predictors: mir184 signature)	Hazard Ratio (95% CI)	P-value
mir184 signature high vs mir184 signature low	1.341 (1.092, 1.648)	**0.0052**

Table S2F Multivariate analysis of prognostic associations for mir184 signature and ER status

Model 6 (Predictors: mir184 signature and ER status)	Hazard Ratio (95% CI)	P-value
mir184 signature high vs mir184 signature low	1.319 (1.043, 1.668)	**0.021**
ER IHC positive vs ER IHC negative	0.935 (0.737, 1.185)	0.58
